# Chromosome 17 copy number changes in male breast cancer

**DOI:** 10.1007/s13402-015-0227-7

**Published:** 2015-04-24

**Authors:** Miangela M. Lacle, Cathy B. Moelans, Robert Kornegoor, Carmen van der Pol, Arjen J. Witkamp, Elsken van der Wall, Josef Rueschoff, Horst Buerger, Paul J. van Diest

**Affiliations:** Department of Pathology, University Medical Center Utrecht, PO Box 85500, 3508 GA Utrecht, The Netherlands; Department of Surgery, University Medical Center, Utrecht, The Netherlands; Department of Division of Oncology, University Medical Center, Utrecht, The Netherlands; Institute of Pathology Nordhessen, Kassel, Germany; Institute of Pathology Paderborn/Höxter and Brustzentrum, Paderborn, Germany

**Keywords:** Male breast cancer, Oncogenes, Copy number changes, Multiplex ligation-dependent probe amplification

## Abstract

**Background:**

Overall, *HER2*-amplified female breast cancer (FBC) is associated with a high grade, an aggressive phenotype and a poor prognosis. In male breast cancer (MBC) amplification of *HER2*, located on chromosome 17, occurs at a lower frequency than in FBC, where it is part of complex rearrangements. So far, only few studies have addressed the occurrence of chromosome 17 alterations in small MBC cohorts.

**Methods:**

Multiplex ligation-dependent probe amplification (MLPA) and fluorescence in situ hybridization (FISH) were used to detect and characterize copy number changes on chromosome 17 in a cohort of 139 MBC. The results obtained were compared to those in FBC, and were correlated with clinicopathological features and patient outcome data.

**Results:**

We observed a lower frequency of chromosome 17 copy number changes with less complex rearrangement patterns in MBC compared to FBC. Chromosome 17 changes in MBC included gains of 17q and losses of 17p. Whole chromosome 17 polyploidies were not encountered. Two recurrent chromosome 17 amplicons were detected: on 17q12 (encompassing the *NEUROD2*, *HER2*, *GRB7* and *IKZF3* gens) and on 17q23.1 (encompassing the *MIR21* and *RPS6KB1* genes). Whole arm copy number gains of 17q were associated with decreased 5 year survival rates (*p* = 0.010). Amplification of *HER2* was associated with a high tumor grade, but did not predict patient survival. Although copy number gains of *HER2* and *NEUROD2* were associated with a high tumor grade, a high mitotic count and a decreased 5 year survival rate (*p* = 0.015), only tumor size and *NEUROD2* copy number gains emerged as independent prognostic factors.

**Conclusions:**

In MBC chromosome 17 shows less complex rearrangements and fewer copy number changes compared to FBC. Frequent gains of 17q, encompassing two distinct amplicons, and losses of 17p were observed, but no whole chromosome 17 polyploidies. Only *NEUROD2* gains seem to have an independent prognostic impact. These results suggest different roles of chromosome 17 aberrations in male versus female breast carcinogenesis.

**Electronic supplementary material:**

The online version of this article (doi:10.1007/s13402-015-0227-7) contains supplementary material, which is available to authorized users.

## Introduction

Previous studies using multiplex ligation-dependent probe amplification (MLPA) and comparative genomic hybridization (CGH) in male breast cancer (MBC) revealed clear differences in gene copy number changes compared to female breast cancer (FBC), pointing towards differences in carcinogenesis between MBC and FBC [1, 2]. Copy number changes on chromosome 17q have been extensively studied in different cancer types including FBC. This is primarily due to the presence of the *ERBB2* oncogene (*HER2*) on chromosome 17q. Amplification of *HER2* is found in about 10–20 % of FBC and usually leads to over-expression of its encoded protein. *HER2* amplification/over-expression also correlates with a high grade, a high mitotic index, a worse prognosis and a favorable response to targeted therapy with trastuzumab [3–8]. Next to *HER2*, several other oncogenes are located on chromosome 17, such as *TOP2A* and *PPM1D* [9–11]. To assess the *HER2* amplification status by in situ hybridization, correction for polysomy of chromosome 17 is widely applied, although several studies have shown that such polysomy is very rare in FBC, and that the copy number status of the centromere does not reliably represent the number of chromosome 17 copies. Instead, it has been found that chromosome 17 may show very complex rearrangements in FBC [12–15]. It has also been found that in MBC *HER2* amplification occurs at a lower frequency than in FBC (2–8 % versus 10–20 %, respectively) [1, 6, 14, 16–18]. So far, only a few (mainly CGH) studies have been published dealing with chromosome 17 alterations in relatively small MBC cohorts [2, 19], and their association with disease outcome has not previously been reported.

In the present study we aimed to characterize copy number changes on chromosome 17 in a large MBC cohort using a dedicated chromosome 17 MLPA kit that has previously been used to study chromosome 17 polysomy in FBC [14]. In addition, we performed *HER2* chromogenic in situ hybridization (CISH) and correlated the results with several clinicopathologic features and patient outcome data.

## Materials and methods

### Patient material and characteristics

Consecutive surgical invasive male breast cancer (MBC) specimens were collected from 1986 to 2011 at four different pathology labs in the Netherlands (i.e., St. Antonius Hospital Nieuwegein, Diakonessenhuis Utrecht, University Medical Center Utrecht, Laboratory for Pathology East Netherlands) as reported in more detail before [1, 20, 21], and at three pathology labs in Germany (i.e., in Paderborn, Cologne, Kassel). Hematoxylin and eosin (HE) stained slides were reviewed by four experienced observers (PJvD, RK, AM, ML) to confirm the diagnosis and to type and grade the tumors according to current WHO standards. Pathology reports were used to retrieve information on age, tumor size and lymph node status. A total of 139 cases, from which the paraffin blocks contained enough tumor cells for DNA isolation, were included in the present study. The clinicopathological features of these cases are listed in Table 1. The average age of the MBC patients was 67 years. The tumor sizes ranged from 0.2 to 7.2 cm. In 114 cases the lymph node status was assessed through axillary lymph node dissection or sentinel node procedures, and in 56 % of these patients lymph node metastases were noted. The majority of the MBC cases was diagnosed (WHO criteria) as invasive ductal carcinoma. According to the modified Bloom and Richardson score [22] most tumors were classified as grade 2 or grade 3. Mitotic activities were assessed as reported before [23], with a mean mitotic index of 12 per 2 mm^2^. In all cases, the estrogen receptor (ER) and progesterone receptor (PR) expression and *HER2* amplification status were re-assessed as described before [20]. Tissue microarray (TMA) slides were used for immunohistochemical detection of ER and PR expression. Chromogenic in situ hybridization (SPoT-Light HER2 CISH kit, Invitrogen) was used for *HER2* copy number assessment. The *HER2* gene was considered to be amplified when more than 50 % of the tumor cells showed 5–10 single dots or small clusters of dots per nucleus (i.e., low level amplification), or more than 10 single dots or large clusters of dots per nucleus (i.e., high level amplification).

**Table 1:** Baseline clinicopathological features of 139 male breast cancers

Characteristics	All cases (*n* = 139)	Characteristics	All cases (*n* = 139)
Age, years		Lymph node metastasis	*n* = 114
Mean	67 (range 32–89)	Absent	50 (44 %)
≤ 50	13 (9 %)	Present	64 (56 %)
> 50	126 (91 %)		
		Immunohistochemistry	
Histological type		ER	
Ductal	124 (90 %)	(+)	128 (92 %)
Lobular	3 (2 %)	(−)	11 (8 %)
Invasive cribriform	3 (2 %)	PR	
Mixed (ductal/lobular)	3 (2 %)	(+)	93 (67 %)
Mucinous	2 (1 %)	(−)	46 (33 %)
Papillary	2 (1 %)	AR	
Invasive micropapillary	1 (1 %)	(+)	112 (81 %)
Adenoid cystic	1 (1 %)	(−)	27 (19 %)
		HER2 (CISH)	
Tumor size (mean), cm	2.3 (*n* = 135)	(+)	5 (4 %)
T1	70 (50 %)	(−)	134 (96 %)
T2	61 (45 %)		
T3	4 (3 %)	Intrinsic subtypes	
		Luminal A	108 (74 %)
Mitotic activity index/2 mm^2^		Luminal B	27 (19 %)
< 8 mitoses	54 (39 %)	Her2 driven	0 (0 %)
8–14 mitoses	34 (24 %)	Basal like /	10 (7 %)
15 or > mitoses	51 (37 %)	unclassified triple negative	
Histological grade			
I	33 (24 %)		
II	60 (43 %)		
III	46 (33 %)		

### Intrinsic subtype classification

Immunohistochemical staining was used to classify the tumors into five intrinsic subtypes: luminal type A (ER+ and/or PR+, HER2- and Ki-67 low), luminal type B (ER+ and/or PR+, and HER2+ and/or Ki67 high), HER2 driven (HER2+ and ER-/PR-), basal-like (ER-/PR-/HER2-, and CK5/6+ and/or CK14+ and/or EGFR+) and unclassifiable triple negative (negative for all six markers), as described before [20].

### DNA extraction and MLPA analysis

Representative tumor areas were identified in HE stained slides and dissected with a scalpel from 8 μm thick paraffin sections as reported before [24]. DNA was extracted by overnight incubation of the samples in proteinase K (10 mg/ml; Roche, Almere, The Netherlands) at 56 °C, boiling for 10 min and subsequent clearance by centrifugation. Five μl of the resulting DNA solution was used for MLPA analysis. MLPA was performed according the manufacturers’ instructions (MRC Holland, Amsterdam, The Netherlands), using a Veriti 96-well thermal cycler (Applied Biosystems, Foster City, CA, USA). A recently designed kit (P004-C1; MRC Holland), containing five probes for five 17p genes and twenty-six probes for seventeen 17q genes, was used. The kit contained twelve additional reference probes. All tests were performed in duplicate. Negative reference samples (normal breast and blood cells) as well as a positive control sample (tumor sample with high level *HER2* amplification as determined by CISH) were included in each MLPA run as reported before [1]. The PCR products were separated by electrophoresis on an ABI 3730 capillary sequencer (Applied Biosystems) and the final gene copy number ratios were calculated using Genescan v4.1 (Applied Biosystems) and Coffalyser v9.4 (MRC-Holland) software packages. For genes represented by more than one probe in the kit, the mean of the copy number ratios in duplicate was calculated. Cut-off values were set as reported before, with > 1.3 to 2.0 for gain, > 2.0 for amplification and < 0.7 for loss. Gene copy number increase (or total gain) was defined by values > 1.3, including gain and amplification. Values between 0.7 and 1.3 were considered normal [6, 25]. Whole chromosome arm loss or gain was defined by copy number loss of more than 75 % of all the probes, as reported before using array-CGH [26]. Partial gain on the long arm of chromosome 17 was defined as any probe showing copy number increase. The MBC chromosome 17 copy number data were also compared to chromosome 17 copy number data of 111 FBC cases reported before, but based on a different MLPA design [14].

### Statistical analyses

Statistical calculations were performed using IBM SPSS for Windows version 20.0. Associations between gene copy numbers and clinicopathological features were calculated using the Pearson Chi-square test (or Fisher’s exact test when appropriate) for categorical variables. Grade, tumor size and mitotic count were dichotomized as usual [1, 21]. Unsupervised hierarchical clustering using the statistical program R (www.r-project.org) was performed to identify relevant clusters. We used the maximum distance and Ward’s clustering method, and calculated the stability of the clusters with pvclust, as reported before [1]. Information regarding prognosis and therapy was retrieved from the Integral Cancer registration of the Netherlands (IKNL). Survival data were available from 100 cases with a mean follow-up of 5.6 years. Therefore, we based the survival analyses on 5 year survival rates. For univariate survival estimates, Kaplan-Meier plots were analyzed using the log rank test. Multivariate survival analyses were performed using Cox regression, including the variables that were found to be significant in univariate analyses. Corrections for multiple comparisons were applied according to Holm-Bonferroni.

## Results

### Identification of chromosome 17 copy number changes in male breast cancers

**Fig. 1:**
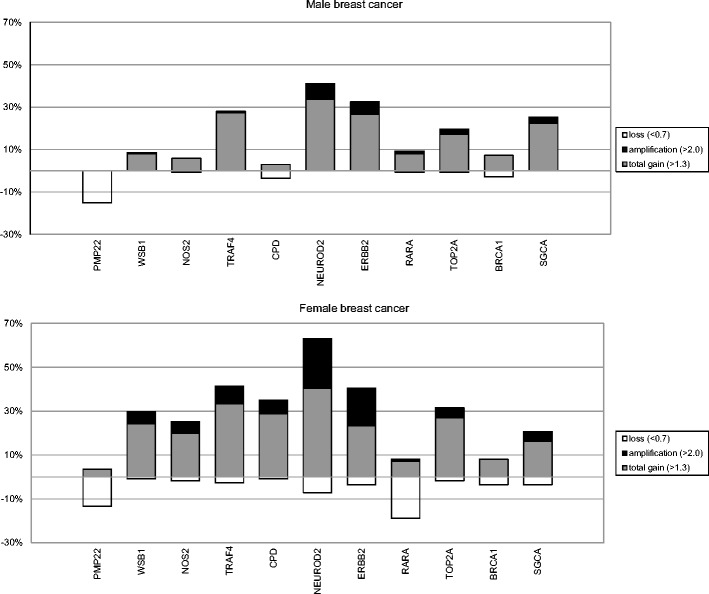
Copy number changes detectecd by MLPA of 11 genes on chromosome 17 in 139 male breast cancers compared to 111 female breast cancers (female data derived from [14])

**Fig. 2:**
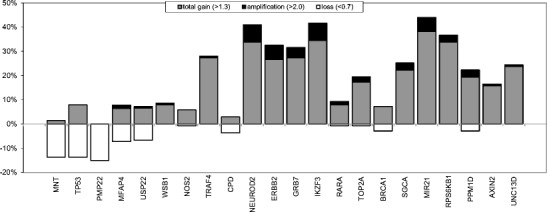
Copy number changes detected by MLPA in 22 genes on chromosome 17 in 139 male breast cancers

Multiplex ligation-dependent probe amplification (MLPA) was used to assess the copy number status of chromosome 17 in a cohort of 139 male breast cancers (MBC). The results obtained and its comparison to female breast cancer (FBC) cases are presented in Fig. 1 and supplementary Table A. Overall, we found a lower frequency of copy number changes with less complex patterns in MBC compared to those in FBC. In 51/139 (36.7 %) cases no copy number alterations were seen in MBC in any of the genes included in the MLPA assay. Copy number increases were found to be most frequent on 17q, present in 78/139 (56 %) of the cases, and copy number losses were found to be most frequent on 17p (36/139; 26 %). Six of the 139 MBC cases (4 %) showed a whole 17q arm gain. None of the MBC cases showed a whole chromosome 17 gain. *NEUROD2*, *IKZF3*, *HER2* and *MIR21* were the most commonly amplified genes, and copy number gains were most common for the *MIR21* and *RPS6KB1* genes. Copy number losses were most frequently observed for the *MNT*, *TP53* and *PMP* genes, all three located on 17p (Fig. 2).

### Chromosome 17 copy number alterations and associations with clinicopathological features

In 8/139 (5.8 %) of the MBC cases the *HER2* status was assessed by MLPA. In four of these eight cases MLPA amplification ratios between 2.0 and 2.5 were observed, whereas the other four showed amplification ratios above 2.5. The cases that exhibited amplification rates between 2.0 and 2.5 by MLPA showed no amplification by CISH, whereas four of the cases with *HER2* amplification as detected by CISH showed amplification by MLPA with ratios > 2.5. One case that was interpreted as showing a low level amplification by CISH also showed a gain, but no high-level amplification, by MLPA (Table 2).

**Table 2:** Her2/Neu status based on immunohistochemistry and CISH in correlation with Her2/Neu amplification status based on MLPA

Her2/Neu immuno- histochemistry	Chromogenic in situ hybridization (CISH) Her2/Neu	Her2/Neu amplification status	Multiplex ligation-dependent probe amplification (MLPA)
Positive (3+)	high amplification	positive	3.795
Positive (3+)	low amplification	positive	4.859
Positive (3+)	high amplification	positive	4.292
Positive (3+)	high amplification	positive	2.536
Negative (2+)	low amplification	positive	1.448*
Negative (0)	no amplification	negative	2.100
Negative (0)	no amplification	negative	2.003
Negative (1+)	no amplification	negative	2.023
Negative (2+)	no amplification	negative	2.269

(*only gain by MLPA, no amplification)

Three of the eight cases (37.5 %) showing *HER2* amplification by MLPA also exhibited a whole arm gain of 17q, including gain of the *WSB1* gene located near the centromere. Two of these cases also showed a partial gain of the short arm, combined with copy number loss of other loci on the short arm. Amplification of the 17q12 region, including the *NEUROD2*, *GRB7* and *IKZF3* genes, was observed in 6/8 (75 %) of the cases with *HER2* amplification, and two of these cases showed an additional amplification of the *RARA*/*TOP2A* gene region on 17q21.2. The *NEUROD2*, *HER2*, *GRB7* and *IKZF3* genes were also frequently found to be gained, whereas copy number loss of this region was observed in none of the cases. Another region of frequent copy number gain was found on 17q23.1, were the *MIR21* and *RPS6KB1* genes are located (Fig. 2). Taken together, we identified two recurrent amplicons: one on 17q12 (encompassing the *NEUROD2*, *HER2*, *GRB7* and *IKZF3* genes) and one on 17q23.1 (encompassing the *MIR21* and *RPS6KB1* genes).

As can be deduced from Table 3, copy number increases of several genes on 17q were correlated with unfavorable clinicopathological features, such as a high mitotic count and a high grade (*NEUROD2*, *HER2*, *GRB7*, *IKZF3*, *RPS6KB1*, *PPM1D*, *AXIN2* and *UNC13D*), a high grade and a large size (*SGCA*), or a high grade alone (*BRCA1* and *MIR21*). Amplification of *NEUROD2* was found to be correlated with a high mitotic count and a high grade. Amplification of the *HER2* and *GRB7* genes was found to be correlated with a high grade, and amplification of the *IKZF3* gene with a high mitotic count. After correction for multiple comparisons, the correlation between copy number gains of the *NEUROD2* gene and a high mitotic count and a high grade remained significant (Table 3).

**Table 3:** Correlations between gene copy number changes and clinicopathological features in 139 male breast cancers

Gene	Location	Mitotic index	Size	Grade	Lymph node status
Correlation between gene copy number increases (>1.3 (including amplified cases)) and clinicopathological features					
* USP22*	17p11.2				0.006
* WSB1*	17q11.1				0.042
* CPD*	17q11.2				0.035
* NEUROD2*	17q12	**<0.0001**		**<0.0001**	
* ERBB2*	17q12	0.046		0.017	
* GRB7*	17q12	0.016		0.006	
* IKZF3*	17q12	**0.001**		0.028	
* RARA*	17q21.2				0.01
* BRCA1*	17q21.31			0.01	0.021
* SGCA*	17q21.33		0.025	0.021	
* MIR21*	17q23.1			0.019	
* RPS6KB1*	17q23.1	**0.002**		**0.001**	
* PPM1D*	17q23.2	0.047		0.033	
* AXIN2*	17q24.1	0.008		**<0.0001**	
* UNC13D*	17q25.1	**<0.0001**		**<0.0001**	
Correlation between gene amplification (>2.0) and clinicopathological features					
* NEUROD2*	17q12	0.049		0.015	
* ERBB2*	17q12			0.016	
* GRB7*	17q12			**0.001**	
* IKZF3*	17q12	0.007			

Blank: non-significant results. Bold: after correction for multiple comparisons

### Cluster analysis reveals association with luminal breast cancer sub-type

After unsupervised hierarchical cluster analysis, we found that the *NEUROD2*, *HER2*, *GRB7* and *IKZF3* genes clustered together (*p* < 0.001) (Supplementary Fig. A). Considering the MBC cases, an interesting cluster emerged encompassing 12 cases characterized by chromosome 17 whole arm copy number gains and amplifications of the *NEUROD2*, *HER2*, *GRB7* and *IKZF3* genes with significantly more luminal type B cases than luminal type A cases (*p* = 0.010). The distribution of other clinicopathological features (age, grade, mitotic index, size and lymph node status) was not found to be significantly different in this cluster compared to the other remaining cases.

### Tumor size and *NEUROD2* copy number gain act as independent prognostic factors

**Fig. 3:**
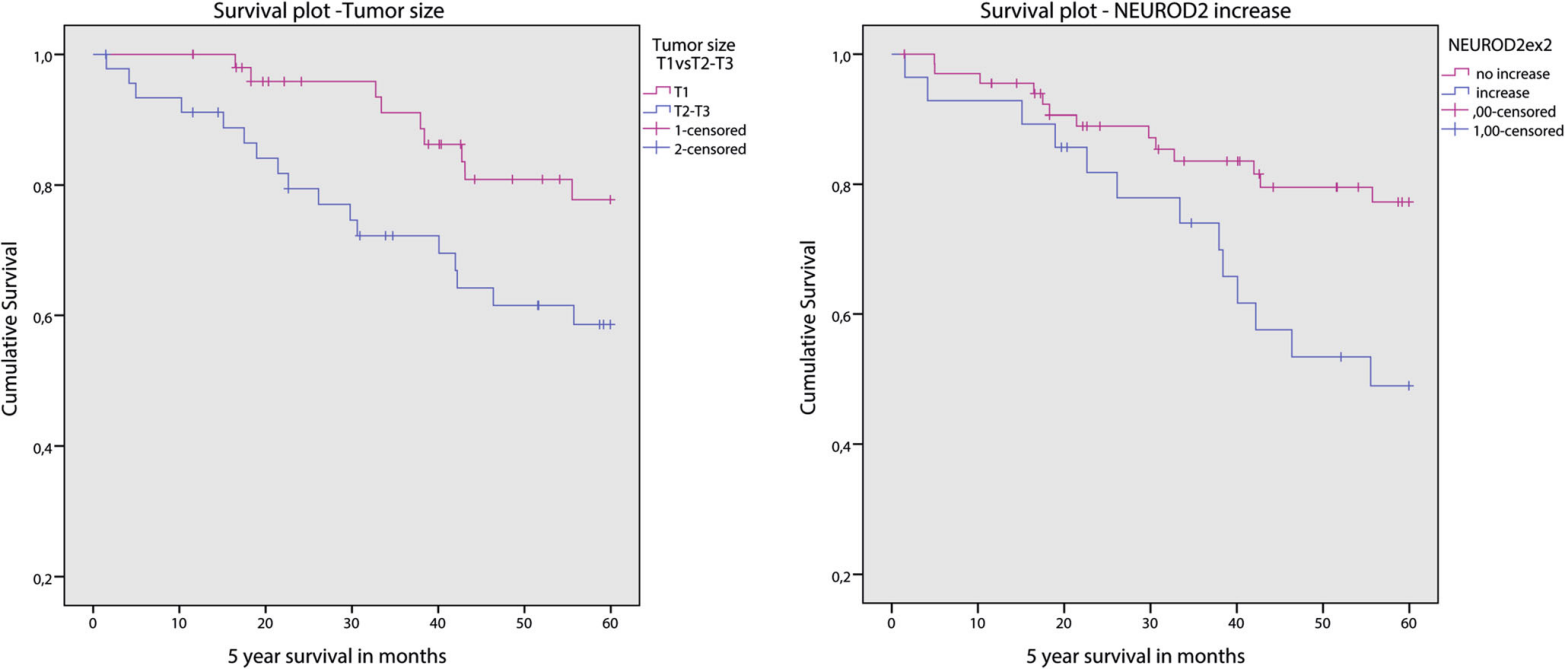
Survival plots of 100 male breast cancers stratified for *NEUROD2* copy number status (*right*) and tumor size (*left*)

Survival data were available from 100 cases with a mean follow up of 5.6 years. Grade 3 (*p* = 0.026), high mitotic count (>8 mitoses/2 mm^2^; *p* = 0.028), large tumor size (>2.0 cm; *p* = 0.031), luminal type B (*p* = 0.042), positive *HER2* status by CISH (low and high level amplification; *p* = 0.039), *NEUROD2* copy number gain (*p* = 0.015), *HER2* copy number gain (*p* = 0.015) and whole chromosome 17q arm gain (*p* = 0.010) were found to be associated with a decreased 5 year survival rate. The above (3.3) described clusters showed no correlation to survival. After multivariate Cox regression only tumor size and *NEUROD2* gene copy number gain remained as independent prognostic factors (Fig. 3).

## Discussion

The aim of the present study was to detect and characterize copy number changes on chromosome 17 in a large cohort of male breast cancers (MBC) using MLPA. The majority of cases showed chromosome 17 aberrations, mainly copy number gains on 17q (78/139; 56 %) and copy number losses on 17p (36/139; 26 %). Only six of the 139 cases (4 %) showed a whole 17q arm gain. None of the cases showed whole chromosome 17 gains, which is in line with previous female breast cancer (FBC) studies [13–15]. Compared to FBC [12], however, we found a lower frequency of chromosome 17 copy number changes with less complex patterns of genomic rearrangements in MBC.

Previously, chromosome CGH was used to assess copy number gains and losses in MBC [2, 19]. Rudlowski et al. [19] reported gains on 17q in 36 % of the MBC tested. In their study 17q gain was not associated with any of the clinicopathological features studied. In the present study we found that 58 % of the MBC showed gains on 17q and 14 % showed high-level amplifications on 17q. In line with our study, Tommasi et al. [2] found that both losses and gains on chromosome 17 were less prominent in MBC than in FBC.

Whole arm gains of 17q and concomitant gains of the *WSB1* gene located near the centromere were frequently seen in association with amplification of the *HER2* gene (37.5 %). The 17p arm, however, only showed partial copy number gains and losses, or no alterations at all in these cases, arguing against the occurrence of whole chromosome 17 polysomies in MBC, as has previously been observed in FBC [12]. These findings suggest that FISH correction for polysomy with centromere probes may result in a misleading *HER2* gene status assessment, as described before in FBC [12].

Although in itself rare, the co-amplification and co-clustering of genes on the *HER2* neighboring segment containing *NEUROD2*, *GRB7* and *IKZF3* in 75 % of the MBC cases points towards the existence of a large amplicon on 17q, which is different from what has previously been seen in FBC [12, 27, 28]. This latter amplicon includes both the *HER2* and *NEUROD2* genes, and is in line with a similar prognostic value of both *NEUROD2* and *HER2* copy number gains in the present study.

We found that the *HER2* gene exhibited copy number gains in 21 % of the MBC cases, but a true amplification (by MLPA) was only seen in 5.8 % of the cases, which is in line with previous *HER2* expression studies [16, 18], but lower than that reported by Tommasi et al. [2], who found *HER2* to be amplified in 30 % of MBC cases by CGH. This discrepancy may be explained by the different technique and smaller sample size used in the latter study. We found that *HER2* was amplified at a lower frequency in MBC compared to previous MLPA studies in FBC (5.8 % versus 20 %) [6]. In concordance with our previous studies in FBC and MBC, *HER2* amplification detected by MLPA correlated strongly with *HER2* amplification detected by CISH [1, 14].

We found that a positive *HER2* status, defined by both CISH (*p* = 0.039) and MLPA (*p* = 0.015), was correlated with a decreased 5 year survival rate. *HER2* amplification was relatively rare (5.8 %) and, by itself, not found to serve as a predictor of survival in univariate survival analyses. This notion may be due to the relatively small number of cases showing *HER2* amplification. Copy number gain of *NEUROD2* was also found to be correlated with a decreased 5 year survival (*p* = 0.015), as was whole 17q arm gain (*p* = 0.010). In multivariate Cox regression analyses, *NEUROD2* copy number gains exhibited an independent prognostic value next to tumor size. *NEUROD2* itself, coding for a protein that plays a role in neuronal differentiation and neuronal cell fate, is unlikely to be involved in breast cancer prognosis. Therefore, other neighbouring genes are more likely to act as drivers of this amplicon. Some candidate genes such as *MED1*, *MED24* and *DARPP-32* are located near the *NEUROD2* gene. *MED1* encodes a subunit of the master transcriptional co-regulator Mediator/TRAP co-activator complex [29] and is a key ERα co-activator [30, 31]. The MED1/MED24 complex was previously found to be frequently and simultaneously over-expressed in FBC and to play an important role in the growth of breast cancer cells via the RAS-mitogen-activated protein kinase (MAPK) pathway [32]. Several studies have indicated that the *MED1* gene may be located within the *HER2* amplicon [27, 28] and that it may play a key role in HER2-mediated tamoxifen resistance [33]. The neighboring *DARPP-32* gene codes for a Dopamine and cAMP-regulated phosphoprotein, and its over-expression has been implicated in resistance to tumor necrosis factor (TNF)-related apoptosis-inducing ligand (TRAIL) receptor targeted therapy in cancer [34]. It would be interesting to further assess their copy number status and prognostic value in MBC.

The second amplicon encompasses *RPS6KB1*, a protein coding gene reported to be amplified and over-expressed in 10-30 % of FBC. We observed *RPS6KB1* copy number gains in 30 % of the MBC cases. Its encoded protein, a ribosomal protein S6 kinase, is positioned downstream of the PI3K and mTOR pathways and is involved in protein synthesis, cellular growth and proliferation, which makes it an interesting target for therapy. Further research is, however, needed to clarify the exact role of *RPS6KB1* copy number changes in MBC.

The *TOP2A* gene encodes topoisomerase 2 alpha, a nuclear protein which plays an important role in DNA replication and mitosis. It is the main target of adjuvant anthracycline-based chemotherapy. *TOP2A* has previously been reported to be frequently amplified in FBC. The prognostic value of *TOP2A* gene amplification in FBC is as yet, however, controversial [9]. We found a lower percentage of *TOP2A* copy number gain in MBC compared to FBC (15 % versus 27 %) [14]. Gain or amplification of *TOP2A* does not seem to be of prognostic value for MBC.

The *PPM1D* gene, previously reported to be frequently amplified in FBC (25 %), was found to be amplified in a low percentage (2.9 %) of MBC, and to be gained in 16.5 % of MBC in our current study. Gain of *PPM1D* showed a trend towards a correlation with high grade and high mitotic count, but did not appear to be a predictor of survival, as has been reported before in FBC [11].

In conclusion, we found that MBC is characterized by copy number gains on 17q, with two distinct amplicons, and copy number losses on 17p. Like in FBC, no whole chromosome 17 polysomy was found. MBC shows a similar, but less complex pattern of chromosome 17 rearrangements and fewer copy number changes than FBC. These results suggest a different role of chromosome 17 in male and female breast cancer development. Whole arm copy number gain of 17q was associated with *HER2* copy number gain. *HER2* and *NEUROD2* copy number gains were found to be associated with a high tumor grade, a high mitotic count and a decreased 5 year survival rate. *NEUROD2* copy number gain seems to serve as an independent prognostic factor, but is unlikely to be a driver of the associated amplicon. Further research is needed to assess copy number changes in neighboring genes, including their putative prognostic/therapeutic role in MBC.

## Electronic supplementary material

ESM 1 (DOCX 335 kb)

ESM 2 (DOCX 19 kb)
