# Supplementary material for: Chromosome 17 copy number changes in male breast cancer
Source: Cell Oncol (Dordr). 2015 Apr 24;38(3):237–45. doi: 10.1007/s13402-015-0227-7 (PMC4445249; doi:10.1007/s13402-015-0227-7)
Supplement: Supplementary file 1 — (DOCX 335 kb) [file 13402_2015_227_MOESM1_ESM.docx]

Supplement figure
