# Supplementary material for: Chromosome 17 copy number changes in male breast cancer
Source: Cell Oncol (Dordr). 2015 Apr 24;38(3):237–45. doi: 10.1007/s13402-015-0227-7 (PMC4445249; doi:10.1007/s13402-015-0227-7)
Supplement: Supplementary file 2 — (DOCX 19 kb) [file 13402_2015_227_MOESM2_ESM.docx]

**Supplement Table.** Copy number changes on chromosome 17 in 139 male breast cancer cases.

(* percentages loss, increase and amplification in female breast cancer derived from our previous Chr 17 FBC paper [[11](#_ENREF_11)])

| **Gene** | **Chromosome** | **# probes** | **%**  **Increase (FBC)**  **>1.3** | |  | **% amplification**  **>2.0** | | **% loss FBC**  **<0.7** | |
| --- | --- | --- | --- | --- | --- | --- | --- | --- | --- |
| *MNT* | 17p13.3 | 1 | 1.4 |  |  | . |  | 13.7 |  |
| *TP53* | 17p13.1 | 1 | 7.9 |  |  | . |  | 13.7 |  |
| *PMP22* | 17p12 | 1 | . | *(3.6)* |  | . | *(.)* | 15.1 | *(13.5)* |
| *MFAP4* | 17p11.2 | 1 | 5.0 |  |  | 1.4 |  | 7.2 |  |
| *USP22* | 17p11.2 | 1 | 5.8 |  |  | 0.7 |  | 6.5 |  |
| *WSB1* | 17q11.1 | 3 | 7.2 | *(24.3)* |  | 0.7 | *(5.4)* | . | *(0.9)* |
| *NOS2* | 17q11.1 | 1 | 5.8 | *(19.8)* |  | . | *(5.4)* | 0.7 | *(1.8)* |
| *TRAF4* | 17q11.2 | 1 | 26.6 | *(33.3)* |  | 0.7 | *(8.1)* | . | *(2.7)* |
| *CPD* | 17q11.2 | 1 | 2.9 | *(28.8)* |  | . | *(6.3)* | 3.6 | *(0.9)* |
| *NEUROD2* | 17q12 | 1 | 26.6 | *(40.5)* |  | 7.2 | *(22.5)* | . | *(7.2)* |
| *ERBB2* | 17q12 | 4 | 20.9 | *(23.4)* |  | 5.8 | *(17.1)* | . | *(3.6)* |
| *GRB7* | 17q12 | 2 | 23.0 | *.* |  | 4.3 | *.* | . |  |
| *IKZF3* | 17q12 | 1 | 27.3 | *.* |  | 7.2 | *.* | . |  |
| *RARA* | 17q21.2 | 1 | 6.5 | *(7.2)* |  | 1.4 | *(0.9)* | 0.7 | *(18.9)* |
| *TOP2A* | 17q21.2 | 3 | 15.1 | *(27.0)* |  | 2.2 | *(4.5)* | 0.7 | *(1.8)* |
| *BRCA1* | 17q21.31 | 2 | 7.2 | *(8.1)* |  | . | *(.)* | 2.9 | *(3.6)* |
| *SGCA* | 17q21.33 | 1 | 19.4 | *(16.2)* |  | 2.9 | *(4.5)* | . | *(3.6)* |
| *MIR21* | 17q23.1 | 1 | 32.4 |  |  | 5.8 |  | . |  |
| *RPS6KB1* | 17q23.1 | 1 | 30.9 |  |  | 2.9 |  | . |  |
| *PPM1D* | 17q23.2 | 1 | 16.5 |  |  | 2.9 |  | 2.9 |  |
| *AXIN2* | 17q24.1 | 1 | 15.1 |  |  | 0.7 |  | . |  |
| *UNC13D* | 17q25.1 | 1 | 23.0 |  |  | 0.7 |  | . |  |
|  |  |  |  |  |  |  |  |  |  |
